# The Impact of Digital Transformation on Corporate Environment Performance: Evidence from China

**DOI:** 10.3390/ijerph191912846

**Published:** 2022-10-07

**Authors:** Lei Wang, Shibo Liu, Wanfang Xiong

**Affiliations:** 1 School of Economics, Huazhong University of Science and Technology, Wuhan 430074, China; 2 School of Management and Economics, The Chinese University of Hong Kong, Shenzhen, 2001 Longxiang Avenue, Longgang District, Shenzhen 518172, China; 3 School of Accounting, Guangzhou Higher Education Mega Center, Guangdong University of Foreign Studies, Panyu District, Guangzhou 510006, China

**Keywords:** digital transformation, environmental pollution, total factor productivity, internal corporate governance, green innovation

## Abstract

In recent years, the rate of climate change appears to have accelerated, and digital transformation and environmental performance have become increasingly important in the field of corporate social responsibility. Previous studies have mainly focused on the economic consequences of digital transformation. However, research on the effect of digital transformation on reducing firms’ emissions is relatively rare. This study focused on two kinds of typical environmental pollutants: waste gas emissions and wastewater emissions. Using data on Chinese listed firms from 2010 to 2018 and adopting the fixed effect model to investigate the emission reduction effect and mechanism of digital transformation on waste gas emissions and wastewater emissions of firms, we found the following: (1) digital transformation significantly reduces pollution emissions; (2) the relationship is more pronounced in state-owned enterprises (SOEs), high-polluting enterprises, and economically developed regions; (3) to gain a more in-depth understanding of how digital transformation affects the pollution emission behavior of firms, we further conducted mechanism tests and found that digital transformation reduces pollution by increasing total factor productivity and green innovation and improving firms’ internal controls. The above conclusions still hold after a series of robustness tests, including alternative econometric specifications and overcoming potential endogeneity with an instrumental variable. Overall, our findings provide new insights into the effect of digital transformation on environmental pollution emissions. Hence, all governments should pay more attention to digital transformation for sustainable development and improved environmental quality.

## 1. Introduction

Given that environmental pollution has a significant negative influence on human health, with various economic consequences, such as reduced life expectancy, infant mortality, and productivity, it has become one of China’s most contentious issues (Kahn et al., 2015) [1,2]. The significance of environmental preservation has brought emission reductions to the attention of governments and the media throughout the world. High-carbon-emission enterprises in nations such as the United States and China in particular are under increased global pressure to significantly cut carbon emissions resulting from energy consumption in their manufacturing operations [3,4]. As global and national guidelines and legislation send a consistent message about reducing emissions, corporate leaders must consider how to address climate change.

The digital economy supports China’s economic growth. From a technological standpoint, a firm’s digital transformation is defined as the integration of digital technologies into its operational aspects. Vial (2021) [5] and Kane et al. (2015) [6] emphasize digital transformation as a process by which digital technologies cause disruptions, eliciting strategic responses from enterprises seeking to change their value-generating routes. The continuing automation of old manufacturing and industrial operations utilizing current smart technologies is referred to as the Fourth Industrial Revolution (Industry 4.0) [7,8]. For instance, the integration of Internet of Things (IoT) contributes to increased automation, improved communication, and self-monitoring, etc. When integrated with the circular economy, Industry 4.0 provides two industrial paradigms that enable new natural resource strategies [8,9].

To what extent does digital transformation affect firms’ environmental performance? This question is of great practical significance as it brings into focus sustainable economic and environmental development.

The relationship between digital transformation and sustainability has attracted wide attention from scholars and regulators. Existing literature focuses on the economic effects of digital transformation, such as stock price crash risk [10], information environment [11], and economic growth [12]. Alakeson and Wilsdon (2002) [13] first advocated for policy development to capitalize on how digital technology might boost economic growth while easing environmental pressure. In this context, the interaction between digitization and sustainability opens exciting possibilities for a greener society and economy, thus advancing the Sustainable Development Goals (SDGs). 

Research on the environmental consequences of digital transformation is still in its infancy. Specifically, Chen and Hao (2022) [14] examine the interplay between digital transformation and how board structure can improve environmental performance. Further, Cheng et al. (2021) [15] find that new energy-efficient technologies can reduce carbon dioxide (CO2) emissions. This is echoed by Kurniawan et al. (2022) [16], who find that digitalization of non-biodegradable waste promoted waste avoidance up to 65%. The literature has addressed the concerns of sustainable development goals (SDGs) in general (e.g., Chen and Hao.,2022; Cheng et al., 2021; Kurniawan et al., 2022; Balogun et al., 2020; ElMassah and Mohieldin., 2020). However, there is currently little evidence that digital paradigms contribute to environmental performance from firm-level empirical studies. The lack of research on the association between digital transformation and environmental performance hinders a comprehensive recognition of the challenge faced by firms in this digital era. In addition, detailed research on digital transformation and pollution emissions might provide insights on how to tailor specific strategies to improve environmental performance.

Given that industrial firms are the main producers of pollution, an assessment of the causal effects of digital transformation on firms’ pollution should be of special interest to economists and regulators concerned with how to minimize pollution emissions. To fill this gap, we try to explore the impact and mechanism of digital transformation on emission reduction of listed firms in China.

In response to these calls, notably those issued by Alakeson and Wilsdon (2002) [13], Chen and Hao (2022) [14], and Cheng et al. (2021) [15], we devised this study to determine whether digital transformation affects firms’ environmental performance. The main conclusions are as follows: First, the digital transformation of enterprises can increase total factor productivity, green innovation, and internal-controls governance, thereby reducing the emissions of firms. Moreover, we show that in SOEs, heavy-polluting enterprises, and developed eastern regions, the impact of digital transformation on firms’ environmental pollution emissions is stronger. 

Our study may complement and extend the research on firms’ environmental emissions in the following ways. First, to the best of our knowledge, we are the first to study the effects of digital transformation on comprehensive environmental performance in China using listed firm-level data. Although research exploiting and correlating digital transformation with various economic outcomes has increased, little is known about the role of changes in digital transformation in environmental pollution emissions. Previous studies have primarily tested whether digital transformation and corporate sustainability form the moderating role of board characteristics [14]. However, we provide a novel explanation for the listed firms based on various environmental indicators from the perspective of digital transformation. Our study differs from existing studies in the following ways: (1) we concentrate on the influence of changes in digital transformation, which is a critical but understudied component impacting harmful emission activities. (2) Our analysis is based on the setting of China’s shift in assessing the necessity of full-scale digitization of listed firms. (3) We also address potential ways that adjustments in digital transformation might lower hazardous emissions from corporations. We focus on three kinds of underlying mechanisms including total factor productivity, internal corporate governance, and green innovation, and use firm-level data to explore the impact of digital transformation on environmental pollution. Overall, we provide new insight into the literature on firms’ digital transformation and enrich the research on the influences on firms’ environmental performance. 

Second, our study contributes to the extensive literature on the determinants of toxic emissions. Previous research has found that local political promotion, economic institutions, and the law can affect environmental pollution [17,18]. However, our study enriches the literature on how digital transformation can affect firms’ environmental pollution. Our findings also provide a new perspective and different policy implications for other emerging markets concerned with environmental quality. Overall, our study fills a research gap and provides fresh insights into reducing firms’ emissions.

The rest of our paper is organized as follows. In Section 2, we provide a literature review and develop the hypotheses. In Section 3, we discuss the data and variables. In Section 4, we present the baseline estimation results and provide a series of robustness checks. In Section 5 and Section 6, we discuss the results of our mechanism analysis and cross-sectional analysis, respectively. Finally, in Section 7, we provide our conclusions and outline policy implications.

## 2. Hypotheses Development

Many scholars believe that digital transformation can drive corporate sustainable development and bring many benefits to business and society. For instance, Kunkel and Matthess (2020) [19] conclude that the potential of information and communication technologies (ICTs) for environmental sustainability are becoming increasingly relevant as their application in industrial production grows. Additional digital applications with the potential to mitigate air pollution have been proposed as part of the SynchroniCity project (funded by Horizon2020). Autonomous air quality management (AAQM), for example, strives to provide tailored solutions to enhance air quality in public settings. Chowdury et al. (2019) [20] documented that several ICT companies, including Microsoft and Nokia, are currently researching how to include IoT-based water-quality monitoring into their approaches to smart cities. In addition, according to the Global Climate Action Summit’s most recent Index Climate Action Roadmap, digital technology solutions in the areas of energy, manufacturing, agriculture, services, transportation, and traffic management could reduce carbon emissions by 15% globally by 2020. 

Furthermore, firms are under pressure from a variety of stakeholders (government, community, media, etc.) who are beginning to focus on environmental concerns. As a result, firms are being obliged to implement proactive environmental governance measures. Cronkleton et al. (2008) [21] found that environmental governance skills of enterprises are particularly appealing to stakeholders and may be promoted by appropriate firms. Thus, corporate environmental management uses a continuous improvement technique to monitor and control their economic impact on the environment to cater to stakeholders’ requirements [22,23].

In summary, the above discussions led to our hypothesis below:

**H1.**  *Digital transformation can improve corporate environmental performance.*

As the digital economy significantly facilitates social productivity through high-tech innovation [24,25], it is considered an innovation driver for TFP [26]. Digital transformation boosts operational agility and production flexibility [27]. On the one hand, manufacturing companies can modify their production plans in response to changes in consumer demand for environmentally friendly products. On the other hand, digital transformation lowers transaction costs and boosts investment effectiveness, which causes high-polluting businesses to scale back on their production. Thus, we expect that:

**H2.**  *Digital transformation can affect pollution emissions through improving enterprise efficiency.*

Furthermore, digital technology can increase information processing and company responsiveness [28]. It makes corporate management and operations more transparent and helps stakeholders understand the company’s environmental governance commitment [29]. Digital technology can enable business managers to have real-time data on production systems, supply networks, and customer consumption to promote sustainable development, according to (Fernando et al., 2019) [30].

Digital technologies are expected to improve corporate environmental management on a product and process level by improving data quality and accessibility (such as real-time machine consumption information) [22]. Chen and Zhang (2022) [11] conclude that digital transformation enhances data timeliness and transparency, while lowering information supply costs. In addition, digital transformation and upgrading include all facets of a company’s activities, such as corporate governance and corporate profitability. Corporate digital transformation can explore and mine data based on digital technology, boost the vitality of the internal data system, and help businesses identify their current production process by lowering the production of harmful substances. Based on the above discussions, we propose Hypothesis 3:

**H3.**  *Digital transformation can affect corporate environment performance through improving internal controls.*

Under the strain of environmental protection, firms view green innovation as a key approach for gaining a sustainable competitive advantage [30]. To improve environmental performance, many Chinese firms have adopted green innovation as a strategic activity [31]. Green innovation may be further broken down into green management innovation and green technology innovation from the perspectives of technology and management procedures [32].

Costa and Matias (2020) [33] demonstrate that digital transformation may create a sustainable innovation ecosystem. Similar findings were made by [34], who discovered that digital technology can result in a more resource-efficient economic system by easing environmental stress. According to [35], green innovation can reduce and even stop the production of pollutants and enhances corporate environmental performance [36]. As a result, green innovation is a key strategy for coordinating the growth of the economy and the improvement of the environment [37].

Thus, we provide Hypothesis 4:

**H4.**  *Digital transformation can affect corporate environment performance through improving green innovation.*

**Figure 1:**
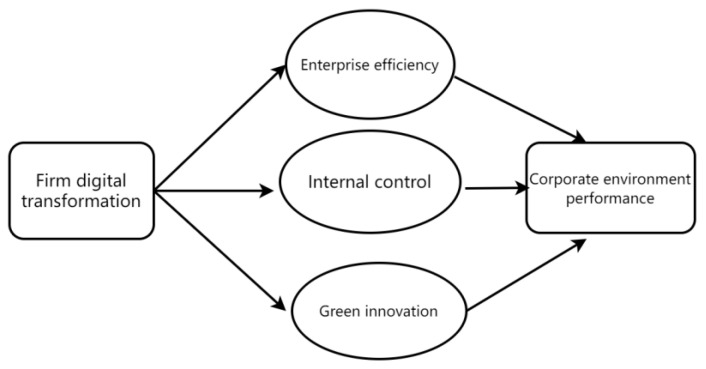
The relationship between digital transformation and corporate environment performance.

Based on the discussions above, we propose our conceptual framework to shed light on the relationship between digital transformation and corporate environmental performance. The framework is presented in Figure 1.

## 3. Econometric Specification and Data

### 3.1. Econometric Specification

Our estimation strategy comprises the following steps. Firstly, we used a fixed effect (FE) panel regression to test H1, on whether digital transformation can explain environmental pollution. The choice of a fixed effect regression over a random effect regression was based on the Hausman test, not detailed here to save space. To test whether digital transformation plays a role in influencing environmental pollution, we estimated the following OLS model:

(1) $$Pollution_{i,t}=\alpha +\beta Digital_{i,t}+\gamma Control_{i,t}+Year_{t}+Industry_{i}+\varepsilon_{i,t}$$

where subscripts i and t represent industry and year, respectively. $Pollution_{i,t}$ is a variable for corporate environmental performance in year t. The variable of interest is $Digital_{i,t}$, which is defined as the logarithm of the number of keywords. Standard errors are robust according to heteroskedasticity and are clustered at the firm level. The fixed effect of industry addresses the concern that the results are driven by different industries or sector-related changes in environmental pollution. Appendix A presents a vector of controls. A negative and significant β is consistent with H1. 

Secondly, we tested H2, which predicts digital transformation boost corporate efficiency by estimating the following regression:

(2) $$\mathrm{Tfp}\_{\mathrm{tp}}_{i,t}=\alpha 1+\beta 1Digital_{i,t}+\gamma 1Control_{i,t}+Year_{t}+Industry_{i}+\varepsilon_{i,t}$$

where $\mathrm{Tfp}\_{\mathrm{tp}}_{i,t}$ is calculated using the LP approach, based on earlier research [38]. The control variables are consistent with the previous section. According to H2, we expect β1 to be positive.

Thirdly, H3 proposes that could improve the environment by strengthening corporate governance, thereby strengthening the supervision of enterprises, and reduce environmental pollution. We establish the following regressions:

(3) $$\mathrm{Internal}\_{\mathrm{control}}_{i,t}=\alpha 2+\beta 2Digital_{i,t}+\gamma 2Control_{i,t}+Year_{t}+Industry_{i}+\varepsilon_{i,t}$$

where $\mathrm{Internal}\_{\mathrm{control}}_{i,t}$ is measured by the internal control index, which is developed by Xiamen University in China. This index is published annually in the three most influential financial newspapers in China: *China Securities Journal*, *Shanghai Securities News*, and *Securities Times*. The index is widely used and cited by the media, auditors, listed companies, and scholars in China. Other variables are defined as in Equations (1) and (2) according to H3, and should be significantly positive. 

Fourthly, H4 proposes that the environment could be improved by strengthening green innovation, thereby strengthening green clean technology to enhance firms’ environmental performance. We establish the following regressions:

(4) $$Patent\_green_{i,t}=\alpha +\beta 3Digital_{i,t}+\gamma 3Control_{i,t}+Year_{t}+Industry_{i}+\varepsilon_{i,t}$$

where $\mathrm{Patent}\_{\mathrm{green}}_{i,t}$ denotes the logarithm of the number of green patents of a firm in a fiscal year. According to Wang and Zhao (2019), green patents provide the intrinsic benefit of measuring green technology innovation. According to the study of Pan et al. (2021) [39], and the World Intellectual Property Organization’s (WIPO) green list of international patent classification launched in 2010, patent information related to environmentally friendly technology is screened, searched, and matched in the State Intellectual Property Office. Other variables are defined as in Equations (1)–(3). According to H4, β3 should be significantly negative. Furthermore, ${\mathrm{Industry}}_{i}$ represents industry fixed effects to control industry-specific factors, ${\mathrm{Year}}_{t}$ is the year fixed effects that account for macro or technological shocks to the economy by treating all industries identically, and $\varepsilon_{i,t}$ is an idiosyncratic error that is assumed to be independent and identically distributed with zero mean and fixed variance.

### 3.2. Variables and Summary Statistics

The study collected data of A-share firms listed on the Shenzhen Stock Exchange and the Shanghai Stock Exchange from the China Stock Exchange and Accounting Research (CSMAR) database. The CSMAR database is comprehensive and appropriate for publicly available Chinese companies. We excluded missing value samples, ST, PT, and other samples. We also excluded the financial industry sample. Finally, data were used from 2008 to 2018 and included 22,635 observations. All continuous variables are winsorized at the 1 percent level.

#### 3.2.1. Digital Transformation

The primary independent variable in this study is digital transformation. The literature on digital transformation measurement focuses mostly on the macro level, such as regional digital transformation, industrial digital transformation, and national digital transformation. Micro-digital transformation measurement is relatively rare. The share of intangible assets connected to digitization is primarily used in the current study [40]. ERP system application [41] was calculated. However, most of these indicators have varying degrees of flaws and shortcomings, which made it difficult to fully and correctly assess digital transformation. To alleviate this load, we developed an index using the text analysis approach based on machine learning pioneered by Wu et al. (2022) [10] and Llopis et al. (2021) [42]. 

Specifically, we used a textual analysis approach to capture the frequency of corresponding digital transformation keywords in annual reports issued by listed firms. We constructed five relevant keywords to describe digital transformation: digitization, artificial intelligence, big data, Internet of Things, and cloud computing. Based on the Word Embedding Model, we end up with 197 terms from the five keywords, including intelligent computing, robotics, and cloud services, into the “jieba” Chinese lexical database of Python package. Then, by analyzing the text of the “Management Discussion and Analysis” (MD&A) section based on machine learning, we obtained the frequency of the 197 digitization-related words in the annual reports of listed companies. Considering the difference in the length of MD&A text of the annual report, we measured the degree of digitization by using the sum of the frequency of words related to enterprise digitization divided by the segment length of the MD&A section of annual report. The larger the value of the digital transformation indicator, the higher the degree of enterprise digitization.

#### 3.2.2. Environmental Pollution Emissions

We focused on two kinds of typical environmental pollutants: waste gas emissions and wastewater emissions. We measured toxic emissions including sulfur dioxide and wastewater at firm level from the CSMAR database. In particular, we collated the detailed pollution emissions of firms, including sulfur dioxide, industrial waste gas, soot, and wastewater. We measured firms’ pollutant emissions by calculating waste gas emissions as a percentage of total output value. 

#### 3.2.3. Control Variables

Following prior literature on firms’ pollution emissions, we extracted an additional set of control variables for consideration as potential factors affecting firms’ environmental performance. The common control variables are the log of the total assets (*Size*); debt-to-assets ratio (*Leverage*); profitability (*ROA*); property, plant, and equipment (*PPE*); growth of operating income (*Growth)*; the ownership ratio of the actual controller (*Nership*); number of Independent Directors on the board (*Indepboard*); shares held by senior management to total share capital (*Share*); an indicator variable that equals 1 if the CEO is the Chairman of the board, and 0 otherwise (*Duality*); ownership proportion of the actual controller (*Wedge*). Appendix A provides detailed definitions of the variables.

Table 1 provides descriptive statistics for the variables of our main regression based on the sample of Chinese public firms from 2010 to 2018. The mean value of Digital is 0.068; the mean of the three kinds of environmental pollution of a firm’s wastewater and waste gas are 0.184 and 0.161, respectively. Firms in our sample have an average Digital of 0.068.

## 4. Empirical Results

### 4.1. Baseline Regression

To test whether digital transformation plays a role in influencing environmental pollution, we estimate the OLS model in Equation (1). Table 2 presents the regression results of testing H1. Columns 1 and 2 use wastewater and waste gas emissions as the environmental pollution quality measures, respectively. Consistent with H1, after controlling for a host of variables, as well as controlling for industry and year fixed effects, the results show a negative relationship between these aspects of digital transformation, with the statistical significance of wastewater and waste gas emissions at the 5% level. Examining the firm-level control variables, the estimated coefficients of size and growth are significantly positive, indicating that greater size and growth are associated with producing more environmental pollution.

### 4.2. Robust Check: Different Model

Considering the other industry-related omitted variables, we further control the interaction term of industry and year fixed effects, and the results remain as reported in column (1) and (2) in Table 3. Our results still hold. 

### 4.3. Robustness Check: 2SLS Estimation

We further tried to establish the causal relationship between digital transformation and firms’ environmental pollution emissions based on the instrumental variable method. Following Wu et al. (2021) [10], we adopted the Internet data as an instrumental variable (IV), which is defined as the cross product of the number of Internet accesses nationwide with a lag of one period and the number of fixed telephones per 10,000 people in each prefecture-level city in 1984, respectively. On the one hand, the development of local telecommunications infrastructure will affect the application of Internet technology in the subsequent stage, both technically and in terms of usage patterns, to meet the relevance requirement. On the other hand, the influence of traditional telecommunications instruments such as landline telephones on economic development steadily declines as use frequency declines, satisfying the exclusivity requirement. The two-stage least squares approach was utilized. 

Columns 1-2 of Table 4 present the estimated results based on instrumental variables regressions. In the first stage, the IV is positively and significantly correlated with *Digital*. The Cragg–Donald f-test statistic from the relevance test of the instrument is 81.27, which is larger than the cutoff value of 10 for a weak instrument hypothesis (testing for the relevance of the IV in the first stage) by Stock and Yogo (2002) [43]. Based on the rule of thumb, we reject the null hypothesis that the instrument is weak. The positive and significant correlations and the high F-statistics in the first stage of estimation indicates that the IV is strongly correlated with digital transformation and serves as a valid instrument.

In the second stage of the 2SLS regression (columns 2–3 of Table 4), the dependent variable is firm environment pollution, and the coefficient for digital (instrumented by IV1) is negatively and statistically significant at the 1% level. Therefore, the results of the 2SLS analysis are consistent with the baseline OLS regression results in Table 4. 

## 5. Mechanism Analysis

### 5.1. Enterprise Efficiency

Here, we discuss the effect of digital transformation on total factor productivity (TFP). Existing research suggests that digital technology is a process that encourages firms to develop new value-creation strategies [5]. These implications contribute to an enterprise’s total factor productivity (TFP). Relich (2017) [44] discovered that ICT software application always enhances labor productivity. This complimentary process has recently been empirically demonstrated in the literature. Pieri et al. (2018) [45], for example, observed the combined effects of R&D and ICT on production. To test H2, we replaced the dependent variable of the baseline regressions with TFP variables. Following Levinsohn and Petrin (2003) [38], we measured this variable by using enterprise TFP estimated by the LP method. We introduced TFP as a proxy variable of enterprise efficiency to examine the mechanism concerning how digital transformation affects firms’ emissions. The results are presented in column 1 of Table 5, showing that the coefficients of digital transformation are positively significant at the 1% level. These results suggest that digital transformation drives TFP performance, which helps verify the potential mechanisms.

### 5.2. Internal Corporate Governance 

Here, we discuss the effect of digital transformation on firms’ internal controls. As we discussed in previous section, digital transformation and upgrading encompass all aspects of a company’s operations, such as corporate governance and profitability. To test H3, we replaced the dependent variable of the baseline regressions with internal control variables. We employed internal controls to measure internal corporate governance. Our measurement for internal controls uses the internal control index developed by Xiamen University in China. This index has been published annually in the three most influential financial newspapers in China: *China Securities Journal, Shanghai Securities News*, and *Securities Times*. The index is widely used and cited by the media, auditors, listed companies, and scholars in China. Kahn et al. (2015) [46] conclude that firm digitization has a positive effect on internal controls and the level of information disclosure. The results are presented in column 2 of Table 5, showing that the coefficient of digital transformation is positively significant. These results suggest that digital transformation drives internal controls, which helps verify the potential mechanisms.

### 5.3. Green Innovation

Here, we discuss the effect of digital transformation on firms’ green innovation. The initial driving force for green development and an essential emphasis for fostering the establishment of an ecological civilization is green technological innovation [47]. Digital technology facilitates the entire green production process and eliminates the spatial barrier [48].To test H4, we replaced the dependent variable of baseline regression with green innovation. We defined green innovation as the natural logarithm of the number of green innovation applications plus one. Our measurement for green innovation is from the CSMAR database. We introduced green patent as a proxy variable of green innovation to examine the mechanism concerning how digital transformation affects firms’ emissions. The results are presented in column 3 of Table 5, showing that the coefficients of digital transformation are positively significant at the 1% level. These results suggest that digital transformation drives green innovation, which helps verify the potential mechanisms.

## 6. Cross-Sectional Analysis

### 6.1. The Effects of Firms’ Ownership Structure 

We further tested whether the relationship between digital transformation and firm pollution emissions captures the effect of firms’ structure. The literature shows that SOEs are helpful for aspects of social welfare such as reducing pollution and increasing employment [14,37]. Generally, the executives of SOEs are appointed by the government, and thus are more likely to support the government’s agenda. By contrast, non-SOEs attach more importance to firms’ profitability. Digital technologies could be used for ethical, inclusive environmental policies. Environmental data support evidence-based policy decisions. New technologies that promote transparency and facilitate civic participation could legitimize environmental decisions. Thus, the promotional effect of digital transformation on green innovation is projected to be stronger in the sample of SOEs [49]. As the government pays more attention to environmental pollution, we predict that SOEs will have strong incentives to cater to the government by engaging in more CSR activities due to the monitoring role of institutional investors. 

We define SOEs as a dummy variable equal to one if the firm is an SOE, and zero otherwise. We split the sample into SOE and non-SOE groups, respectively. The results are presented in columns (1)–(4) of Table 6. They show that the coefficients for Digital are significant at the 1% level for the SOE subsample but insignificant for the non-SOE subsample. Our results show that the effect of digital transformation on firms’ environmental performance is generally more pronounced for SOEs than for non-SOEs. 

### 6.2. The Effect of Different Regions

A striking feature of China’s pollution situation is the significant difference in pollution levels in different regions. To examine the situation at the regional level, we divided the samples according to whether the enterprise belongs to the eastern region or to the central and western regions. Firms in western China are more polluting and industrially denser, while eastern and central firms have less pollution and lower productivity [36]. 

Table 7 provides a further illustration of cross-sectional differences based on the baseline regression. It can be concluded that for the eastern region, digital transformation has a negative relationship with wastewater and waste gas, negatively significant at the 1% level and 10% level, respectively. It reveals that the coefficients for digitization are not pronounced in underdeveloped areas. Different regions in China differ greatly in terms of infrastructural development and industrial structure. Most of the enterprises in the eastern region are mainly high-end manufacturing industries with lower digital transformation costs and larger technological upgrades, which help to reduce the environmental pollution emissions of enterprises. 

### 6.3. The Effect of Industry Heterogeneity

The different technical talent reserves of heavy-polluting industries and cleaning industries will affect the pace of the digital transformation of enterprises. We further tested whether the relationship between digital transformation and firms’ environmental pollution emissions captures the effect of industry. Polluting enterprises and clean enterprises have significant differences in factor-input structure and environmental adjustment costs. Generally speaking, polluting enterprises face greater pressure for transformation under the vision of “carbon peaking” and “carbon neutrality”, and urgently need to improve factor efficiency and innovation of production technologies. That is, polluting enterprises have greater incentives for digital transformation, which helps the optimization and adjustment of industrial structure.

To test our prediction, we split our sample into heavy-pollution industry groups and cleaning industry groups. The results are presented in column (1)–column (4) in Table 8, showing that the coefficients of *Digital* are negatively significant at the 5% level in the heavy-polluting group and negatively significant at the 10% level in cleaning industry group, which reveals that *Digital* has a larger impact on heavy-polluting firms than on cleaning industry firms. In other words, heavy-polluting firms have a strong incentive to adopt digital transformation and reduce environmental pollution.

## 7. Conclusions and Policy Implications

The digital revolution is significantly influenced by national policies. The digitalization of listed firms is still in its infancy in many nations. Therefore, now could be a key moment to create the groundwork for firm digital transformation that is centered on environmental sustainability. To achieve this, a more active dialogue between scientists and policymakers is required regarding the certainties and uncertainties pertaining to the influence of the digital transformation on environmental sustainability in enterprise. While the volume of scientific knowledge that policy makers must deal with is growing, science itself is still mostly unsure of how the digital transition will affect social development goals.

In this paper, we explored the impact of digital transformation on environmental sustainability. By using Chinese A-share listed firms from 2010 to 2018, this study empirically tests the relationship between digital transformation and enterprise environmental performance. The main conclusion are as follows: first, the digital transformation of enterprises can increase total factor productivity, green innovation, and internal controls governance, thereby reducing the pollution emission of firms. Moreover, we document that in SOEs firms, high polluting enterprises, and developed eastern regions, the impact of the digital transformation of enterprises on the firm environmental pollution emission is stronger. Our findings provide new insights into the impact of enterprise digital transformation on corporate social responsibility that are useful to investors and firms operating in the Chinese stock market. Our study also has a certain significance for emerging market economies regarding how to improve environment performance.

Our study contributes to the research stream on the factors influencing corporate environmental performance by highlighting the link between digital transformation and pollutant emissions. Prior research has shown that digital transformation may be related to the corporate sustainability form the moderating role of board characteristics [14], waste recycling [16], climate change adaption [50], CO_2_ emissions [15], and sustainable development goals (SDGs) [51]. To the best of our knowledge, no study has explored the relationship between digital transformation and firm pollutant emissions (waste gas emissions and wastewater emissions). Our findings are essential for gaining a comprehensive understanding of how digital transformation affect firm environmental performance and will inspire future research exploring the effect of digital transformation.

### 7.1. Managerial Implications

Our work offers relevant policy recommendations for the system of environmental target accountability and suggests a potential way that enterprise digital transformation may influence a firm’s pollution levels. We think that governments around the world that are concerned with digital transformation and environmental conservation should be particularly interested in this topic. In particular, there is ongoing debate on how to properly control and avoid pollution. Although the large-scale closure of energy- and pollution-intensive businesses can help the environment in the short term, it will also have a long-term negative impact on economic growth. Additionally, whereas local governments may not have much incentive to implement environmental controls for the sake of local economic development, the central government is highly motivated to reduce pollution. Our findings demonstrate that the digital transformation offered to local governors can lower business pollution emissions, providing a fresh perspective on the present environmental protection strategy. Therefore, incentivizing local governors to cut pollution by linking the emissions quota to the performance of officials’ assessment systems could be useful.

### 7.2. Practicing Implications

This study has a number of significant practical ramifications. This work primarily responds to a practical request for assistance in comprehending how to employ digitalization to accomplish sustainability-related aims. The results of this study also enrich practitioners’ knowledge on the subject and may provide them with access to the particulars of existing academic output, which have been profiled and examined for the first time in this study. At the same time, the suggested study themes and the supplied research agenda greatly aid in meeting the needs of practitioners, particularly in the use of digitalization for strategic goals.

Based on the above analysis, we propose the following: Our study’s main finding is that corporate digital transformation can reduce pollution. Enterprises must be helped to grasp digitization in order to support corporate digital transformation. Traditional businesses should pursue precise development and increase operational efficiency through digitization while embracing the most recent technologies. Additionally, the businesses affected by digital transformation should receive an appropriate amount of legislative subsidies.

This study can assist people, particularly policymakers, practitioners, and other researchers, in better understanding the current trends in the application of digital transformation and the extent to which this development eases the shift toward greater sustainability. Setting concrete goals for minimizing non-recyclable garbage and setting increased research priorities for the relationship between digitalization and sustainability are necessary in the ongoing transition to a digital economy. The best way to balance rules and incentives should be covered in such studies as well. Instead of viewing the adoption of the digital transformation concept as a benefit in and of itself, it is always important to analyze it critically in light of the SDGs. 

## Figures and Tables

**Table 1:** Variable description. This table presents the firm-year Leverage descriptive statistics for our regression. All variable definitions are presented in Appendix A.

	Obs	Mean	SD	Min	Median	Max
*Digital*	22,635	0.068	0.723	0	0	7.813
*Wwater*	22,635	0.184	1.433	0	0	12.617
*Wgas*	22,635	0.161	0.352	0	0	1.609
*Size*	22,635	22.095	1.311	19.983	21.874	27.285
*ROA*	22,635	0.047	0.049	−0.164	0.043	0.192
*Lev*	22,635	0.399	0.198	0.007	0.392	0.989
*Growth*	22,635	0.633	0.416	0.084	0.536	2.426
*PPE*	22,601	0.045	0.047	0	0.033	0.304
*Nership*	21,956	36.672	16.501	5.042	35.44	75.46
*Wedge*	21,952	4.614	7.431	0	0	27.928
*Duality*	22,363	0.276	0.447	0	0	1
*Restriction*	22,635	0.736	0.621	0.027	0.563	2.884
*Indepboard*	22,633	37.391	5.292	33.33	33.33	57.14
*Compensation*	22,602	0.472	0.131	0.233	0.456	0.866
*Share*	22,005	15.597	21.038	0	1.403	69.337

**Table 2:** Baseline regression. This table reports the results of OLS regressions examining the effect of digital transformation on environmental pollution. Appendix A provides definitions of the variables. The regressions control for a year and Industry fixed effects. In parentheses are *t*-statistics based on standard errors adjusted for heteroskedasticity and firm clustering.

	(1)	(2)
	Wwater	Wgas
*Digital*	−0.028 **	−0.046 **
	(−2.43)	(−2.41)
*Size*	0.041 ***	0.101 ***
	(3.20)	(3.72)
*ROA*	−0.117	0.037
	(−0.79)	(0.13)
*Lev*	−0.049	−0.184
	(−0.89)	(−1.50)
*Growth*	0.055 **	0.102 **
	(2.17)	(2.09)
*PPE*	−0.357 ***	−0.682 *
	(−3.13)	(−1.75)
*Nership*	0.000	0.002
	(0.06)	(1.49)
*Wedge*	−0.001	−0.003
	(−0.76)	(−1.14)
*Duality*	−0.001	−0.034
	(−0.09)	(−1.19)
*Restriction*	0.001	0.031
	(0.11)	(1.15)
*Indepboard*	0.000	0.002
	(0.08)	(0.73)
*Compensation*	−0.061	−0.156
	(−0.98)	(−1.30)
*Share*	−0.001 *	−0.001
	(−1.80)	(−1.05)
*Constant*	−0.775 ***	−2.047 ***
	(−2.77)	(−3.52)
*Year FE*	Y	Y
*Industry FE*	Y	Y
N	21,030	21,030
*R^2^*	0.028	0.052

Superscripts *, **, and *** denote significance at the 10%, 5%, and 1% levels, respectively.

**Table 3:** Robust check: different model. This table reports the results of OLS regressions examining the effect of digital transformation on environmental pollution. Appendix A provides variable definitions. The regressions control for the interaction term of industry and year fixed effects. In parentheses are *t*-statistics based on standard errors adjusted for heteroskedasticity and firm clustering.

	(1)	(2)
	Wwater	Wgas
*Digital*	−0.021 ***	−0.036 ***
	(−3.10)	(−2.91)
*Size*	0.043 ***	0.144 ***
	(6.81)	(9.37)
*ROA*	−0.164	−0.032
	(−1.42)	(−0.15)
*Lev*	−0.119 ***	−0.406 ***
	(−3.67)	(−5.29)
*Growth*	0.052 ***	0.077 ***
	(4.07)	(3.33)
*PPE*	−0.331 ***	−0.156
	(−5.53)	(−0.76)
*Nership*	−0.000	0.002 **
	(−0.69)	(2.53)
*Wedge*	−0.001	−0.003 *
	(−1.64)	(−1.71)
*Duality*	0.009	−0.018
	(0.74)	(−0.92)
*Restriction*	0.008	0.032 *
	(0.96)	(1.76)
*Indepboard*	0.001	0.002
	(0.50)	(1.16)
*Compensation*	−0.063 *	−0.152 **
	(−1.67)	(−2.07)
*Share*	−0.001 **	−0.001 **
	(−2.14)	(−2.05)
*Constant*	−0.742 ***	−2.683 ***
	(−5.24)	(−8.21)
Industry FE * Year FE	Y	Y
*N*	21,031	21,031
*R* ^2^	0.008	0.023

Superscripts *, ** and *** denote significance at the 10%, 5% and 1% levels, respectively.

**Table 4:** 2SLS estimation. This table reports the results of two-stage least-squares (2SLS) regressions with the cross product of the number of Internet accesses nationwide with a lag of one period and the number of fixed telephones per 10,000 people in each prefecture-level city in 1984 as the instrumental variables (IVs). Appendix A provides definitions of variables. The regressions control for one year and industry fixed effects. In parentheses are *t*-statistics based on standard errors adjusted for heteroskedasticity and firm clustering.

	(1)	(2)	(3)
	Digital	Wwater	Wgas
*Digital (instrumented)*		−0.984 **	−5.883 ***
		(−2.48)	(−3.85)
*IV*	0.004 ***		
	(4.15)		
*Size*	0.002	0.037 ***	0.137 ***
	(0.65)	(5.75)	(5.52)
*ROA*	−0.059	−0.212	−0.576
	(−0.67)	(−1.45)	(−1.02)
*Lev*	−0.275 ***	−0.384 ***	−2.075 ***
	(−10.52)	(−3.26)	(−4.57)
*Growth*	−0.051 ***	0.001	−0.200 **
	(−5.39)	(0.05)	(−2.02)
*PPE*	−0.981 ***	−1.161 ***	−5.570 ***
	(−12.04)	(−2.79)	(−3.47)
*Nership*	−0.003 ***	−0.003 **	−0.014 ***
	(−9.96)	(−2.57)	(−3.10)
*Wedge*	−0.002 ***	−0.003 **	−0.016 ***
	(−4.13)	(−2.33)	(−2.94)
*Duality*	0.054 ***	0.032	0.280 ***
	(6.14)	(1.26)	(2.88)
*Restriction*	0.032***	0.033*	0.195 ***
	(4.69)	(1.95)	(3.02)
*Indepboard*	0.005 ***	0.005 **	0.027 ***
	(6.26)	(2.07)	(3.19)
*Compensation*	−0.054 *	−0.110 **	−0.558 ***
	(−1.82)	(−2.04)	(−2.67)
*Share*	0.00 2***	0.002 **	0.010 ***
	(8.42)	(2.04)	(3.32)
*Constant*	0.430 ***	−0.240	−0.051
	(4.94)	(−1.13)	(−0.06)
*Year FE*	Y	Y	Y
*Industry FE*	Y	Y	Y
*N*	18,049	18,049	18,049
*F-test*	81.27		

Superscripts *, **, and *** denote significance at the 10%, 5%, and 1% levels, respectively.

**Table 5:** Channel. This table reports the results of OLS regressions examining the effect of digital transformation on TFP, internal control index, and green innovation. Appendix A provides definitions of variables. The regressions control for one year and industry fixed effects. In parentheses are *t*-statistics based on standard errors adjusted for heteroskedasticity and firm clustering.

	(1)	(2)	(3)
	Tfp_tp	Internal Control Index	Green Innovation
*Digital*	0.040 ***	0.013 **	0.014 ***
	(3.89)	(2.13)	(6.75)
*Size*	0.600 ***	0.001	−0.001 **
	(86.95)	(0.32)	(−2.28)
*ROA*	1.243 ***	−0.043	−0.022 *
	(15.02)	(−0.78)	(−1.93)
*Lev*	0.336 ***	−0.078 ***	−0.041 ***
	(8.82)	(−3.97)	(−10.95)
*Growth*	1.165 ***	−0.009	−0.022 ***
	(54.97)	(−0.95)	(−14.72)
*PPE*	−0.391 ***	0.068	0.053 ***
	(−2.97)	(1.03)	(3.10)
*Nership*	−0.000	0.000 *	−0.000 **
	(−1.46)	(1.73)	(−2.32)
*Wedge*	0.000	0.002 ***	0.000
	(0.12)	(3.61)	(1.00)
*Duality*	−0.001	0.014 ***	0.003 ***
	(−0.17)	(2.90)	(2.78)
*Restriction*	0.000	0.017 ***	0.002 **
	(0.03)	(4.00)	(2.39)
*Indepboard*	−0.001	0.000	0.000 **
	(−1.28)	(1.06)	(2.02)
*Compensation*	0.114 ***	0.002	−0.014 ***
	(3.41)	(0.09)	(−3.05)
*Share*	0.001 ***	0.001 ***	0.000 **
	(5.50)	(5.12)	(2.33)
*Constant*	−6.112 ***	0.862 ***	0.096 ***
	(−38.53)	(13.05)	(7.49)
*Year FE*	Y	Y	Y
*Industry FE*	Y	Y	Y
*N*	18,145	21,030	17,045
*R^2^*	0.928	0.244	0.473

Superscripts *, **, and *** denote significance at the 10%, 5%, and 1% levels, respectively.

**Table 6:** Cross-section analysis: firms’ ownership structure. This table reports the results of OLS regressions examining the effect of digital transformation on environmental pollution. In columns 1–4, we split the samples into SOE and non-SOE groups. Appendix A provides definitions of variables. The regressions control for a year and industry fixed effects. In parentheses are *t*-statistics based on standard errors adjusted for heteroskedasticity and firm clustering.

		Non-SOEs SOEs		
	(1)	(2)	(3)	(4)
	Wwater	Wgas	Wwater	Wgas
*Digital*	−0.035	−0.024	−0.057 ***	−0.028 ***
	(−1.25)	(−0.58)	(−2.93)	(−3.06)
*Size*	0.047 **	0.089 **	0.117 ***	0.039*
	(2.48)	(2.27)	(2.74)	(1.80)
*ROA*	0.022	0.154	0.055	−0.115
	(0.07)	(0.27)	(0.16)	(−0.64)
*Lev*	−0.090	−0.256	−0.150	−0.040
	(−0.80)	(−1.04)	(−1.20)	(−0.66)
*Growth*	0.050	0.118	0.095*	0.058 **
	(1.11)	(1.42)	(1.69)	(2.18)
*PPE*	−0.506 ***	−0.992	−0.291	−0.210
	(−2.94)	(−1.52)	(−0.87)	(−1.32)
*Nership*	−0.001	0.002	0.002	0.001
	(−1.07)	(0.65)	(1.14)	(1.02)
*Wedge*	−0.001	−0.005	−0.002	−0.001
	(−0.42)	(−1.21)	(−0.72)	(−1.18)
*Duality*	−0.008	−0.004	−0.037	0.010
	(−0.28)	(−0.08)	(−1.06)	(0.57)
*Restriction*	−0.027	−0.025	0.052*	0.008
	(−0.88)	(−0.43)	(1.65)	(0.53)
*Indepboard*	−0.002	0.002	0.003	0.002
	(−0.85)	(0.36)	(0.77)	(0.83)
*Compensation*	0.013	−0.210	−0.183	−0.114
	(0.11)	(−0.95)	(−1.27)	(−1.62)
*Share*	−0.000	−0.002	−0.001	−0.001
	(−0.16)	(−1.01)	(−0.45)	(−1.45)
*Constant*	−0.759 **	−1.675 **	−2.468 ***	−0.811
	(−2.03)	(−2.05)	(−2.61)	(−1.61)
*Year FE*	Y	Y	Y	Y
*Industry FE*	Y	Y	Y	Y
*N*	8677	8677	12,352	12,352
*R^2^*	0.038	0.072	0.043	0.037

Superscripts *, **, and *** denote significance at the 10%, 5%, and 1% levels, respectively.

**Table 7:** Cross-section analysis: different origin. This table reports the results of OLS regressions examining the effect of digital transformation on environmental pollution. In columns 1–4, we split the samples into the eastern regions group and the central and western regions group. Appendix A provides definitions of variables. The regressions control for a year and industry fixed effects. In parentheses are *t*-statistics based on standard errors adjusted for heteroskedasticity and firm clustering.

	Eastern Regions		Central and Western Regions	
	(1)	(2)	(3)	(4)
	Wwater	Wgas	Wwater	Wgas
*Digital*	−0.025 **	−0.041 *	−0.019	−0.033
	(−1.99)	(−1.90)	(−0.69)	(−0.87)
*Size*	0.032 **	0.112 ***	0.072 **	0.092
	(2.22)	(3.62)	(2.56)	(1.59)
*ROA*	−0.126	0.000	−0.158	0.369
	(−0.82)	(0.00)	(−0.37)	(0.51)
*Lev*	0.007	−0.133	−0.239	−0.292
	(0.13)	(−1.01)	(−1.59)	(−1.13)
*Growth*	0.019	0.061	0.129 **	0.150
	(0.73)	(1.18)	(2.21)	(1.37)
*PPE*	−0.265 **	−0.289	−0.604 **	−1.606 *
	(−2.13)	(−0.72)	(−2.17)	(−1.79)
*Nership*	−0.000	0.002	0.001	0.003
	(−0.59)	(1.22)	(0.86)	(0.87)
*Wedge*	−0.000	0.000	−0.004	−0.012 *
	(−0.01)	(0.12)	(−1.60)	(−1.79)
*Duality*	−0.001	−0.030	−0.000	−0.052
	(−0.06)	(−0.89)	(−0.00)	(−0.83)
*Restriction*	0.011	0.036	−0.034	−0.002
	(0.79)	(1.16)	(−1.29)	(−0.03)
*Indepboard*	0.002	0.004	−0.006 *	−0.003
	(0.90)	(1.21)	(−1.82)	(−0.50)
*Compensation*	−0.020	−0.095	−0.181	−0.415*
	(−0.29)	(−0.70)	(−1.54)	(−1.68)
*Share*	−0.000	−0.000	−0.003 ***	−0.003
	(−0.63)	(−0.47)	(−2.71)	(−0.95)
*Constant*	−0.671 **	−2.430 ***	−1.103 **	−1.420
	(−1.98)	(−3.53)	(−2.11)	(−1.33)
*Year FE*	Y	Y	Y	Y
*Industry FE*	Y	Y	Y	Y
*N*	15,307	15,307	5721	5721
*R^2^*	0.035	0.059	0.048	0.072

Superscripts *, **, and *** denote significance at the 10%, 5%, and 1% levels, respectively.

**Table 8:** Cross-section analysis: The effect of industry heterogeneity. This table reports the results of OLS regressions examining the effect of digital transformation on environmental pollution. In columns 1–4, we split the samples into clean industry group and heavy-polluting industry group. Appendix A provides definitions of variables. The regressions control for a year and industry fixed effects. In parentheses are *t*-statistics based on standard errors adjusted for heteroskedasticity and firm clustering.

	Cleaning Industry		Heavy Polluting Industry	
	(1)	(2)	(3)	(4)
	Wwater	Wgas	Wwater	Wgas
*Digital*	−0.132 *	−0.200 *	−0.023 **	−0.040 **
	(−1.86)	(−1.68)	(−1.97)	(−2.17)
*Size*	0.030	0.106	0.047 ***	0.101 ***
	(1.21)	(1.53)	(3.37)	(3.65)
*ROA*	−0.423	−0.423	0.002	0.136
	(−1.20)	(−0.56)	(0.01)	(0.56)
*Lev*	−0.061	−0.165	−0.047	−0.163
	(−0.44)	(−0.52)	(−0.86)	(−1.31)
*Growth*	0.170 **	0.232 **	0.025	0.051
	(2.43)	(2.04)	(0.94)	(0.98)
*PPE*	−0.590	−0.857	−0.255 ***	−0.661
	(−1.55)	(−1.02)	(−2.75)	(−1.49)
*Nership*	0.001	0.005	−0.000	0.001
	(0.67)	(1.56)	(−0.60)	(0.61)
*Wedge*	−0.000	−0.002	−0.001	−0.003
	(−0.10)	(−0.40)	(−1.08)	(−0.94)
*Duality*	0.013	−0.155 **	−0.007	0.011
	(0.32)	(−2.15)	(−0.47)	(0.39)
*Restriction*	0.011	0.092	−0.005	0.010
	(0.31)	(1.11)	(−0.44)	(0.47)
*Indepboard*	−0.005	−0.004	0.002	0.004
	(−1.33)	(−0.60)	(0.96)	(1.31)
*Compensation*	0.009	−0.480	−0.092 *	−0.033
	(0.05)	(−1.43)	(−1.82)	(−0.30)
*Share*	−0.001	0.000	−0.001 *	−0.001 *
	(−1.23)	(0.04)	(−1.88)	(−1.79)
*Constant*	−0.421	−1.832	−0.945 ***	−2.182 ***
	(−0.79)	(−1.33)	(−3.04)	(−3.46)
*Year FE*	Y	Y	Y	Y
*Industry FE*	Y	Y	Y	Y
*N*	5969	5969	15,061	15,061
*R^2^*	0.016	0.040	0.034	0.066

Superscripts *, **, and *** denote significance at the 10%, 5%, and 1% levels, respectively.

## Data Availability

The data presented in this study are available on request from the corresponding author.

## Appendix A. Variable Definitions

**Table A1:** Variable Definitions.

*Variables*	Definition
*Digital*	Frequency of occurrence of the corresponding digital keywords in the annual reports published by listed companies as a proxy indicator for the degree of corporate digital transformation
*Wwater*	Natural logarithm of the ratio of industrial wastewater discharge (in 10,000 tons) to the total assets (in billions of RMB) of firm
*Wgas*	Natural logarithm of the ratio of waste gas emissions (in tons) to the total assets (in billions of RMB) of firms
*Size*	Logarithm of total assets (both in millions of RMB)
*ROA*	Net income divided by total assets (both in millions of RMB)
*Leverage*	Debt-to-assets ratio (both in millions of RMB)
*Growth*	Net income/total assets (both in millions of RMB)
*PPE*	Firm property, plant, and equipment scaled by total assets (both in millions of RMB)
*Nership*	Ownership ratio of the actual controller
*Wedge*	Calculated by dividing the ownership ratio by control ratio
*Duality*	Dummy variable that equals 1 if the CEO is the chairman of the board, and 0 otherwise
*Restriction*	Ownership of 2-5 shareholders/ownership of largest shareholder
*Indepboard*	Number of independent directors on the board
*Compensation*	Annual salary of the top three managers/annual management salary (both in thousands of RMB)
*Share*	Total number of shares held by senior management/total share capital
